# Dispersal distance is driven by habitat availability and reproductive success in Northern Great Plains piping plovers

**DOI:** 10.1186/s40462-021-00293-3

**Published:** 2021-12-11

**Authors:** Rose J. Swift, Michael J. Anteau, Kristen S. Ellis, Megan M. Ring, Mark H. Sherfy, Dustin L. Toy

**Affiliations:** U.S. Geological Survey – Northern Prairie Wildlife Research Center, 8711 37th St SE, Jamestown, ND 58401 USA

**Keywords:** Breeding dispersal, Natal dispersal, Shorebird, Density dependence, Conspecific attraction

## Abstract

**Background:**

Dispersal is a critical life history strategy that has important conservation implications, particularly for at-risk species with active recovery efforts and migratory species. Both natal and breeding dispersal are driven by numerous selection pressures, including conspecific competition, individual characteristics, reproductive success, and spatiotemporal variation in habitat. Most studies focus on dispersal probabilities, but the distance traveled can affect survival, fitness, and even metapopulation dynamics.

**Methods:**

We examined sources of variation in dispersal distances with 275 natal dispersal and 1335 interannual breeding events for piping plovers (*Charadrius melodus*) breeding in the Northern Great Plains between 2014 and 2019.

**Results:**

Natal dispersal was on average longer (mean: 81.0 km, median: 53 km) than adult breeding movements (mean: 23.7 km, median: 1 km). Individuals moved the shortest distances when hatched, previously nested, or settling on river habitats. When more habitat was available on their natal area than in the year prior, hatch-year birds moved shorter distances to their first breeding location. Similarly, adults also moved shorter distances when more habitat was available at the settling site and when in closer proximity to other known nesting areas. Additionally, adult movement distance was shorter when successfully hatching a nest the year prior, retaining a mate, or initiating a current nest earlier.

**Conclusion:**

Habitat availability appears to be associated with dispersal distance for both hatch-year and adult piping plovers. Conservation efforts that integrate dispersal distances may benefit from maintaining nesting habitat within close proximity to other areas for adults and a network of clustered sites spread out across a larger landscape for natal dispersal.

## Introduction

Dispersal is a fundamental life-history trait that affects individual fitness, gene flow, and population structure through the movement of individuals between spatial locations [25]. The redistribution of individuals that occurs through dispersal is thus a ‘mobile link’ between breeding areas that occurs less frequently than foraging movements and less regularly than migratory movements [32]. Numerous selection pressures may simultaneously influence dispersal movements including inbreeding avoidance, resource competition, and spatiotemporal variation in habitat suitability or availability [12, 33, 60]. Dispersal is generally divided into two age-based categories: natal dispersal, which is defined as the movement from an individual’s natal area to their first breeding territory, and breeding dispersal, defined as the movement between successive breeding territories [25, 42]. Despite its importance, drivers of variation in dispersal probability and distance are less well studied than other animal movements such as migration and provisioning [22, 28, 63]. While technological advances in gps biologgers that pass data to satellite or cellular networks have greatly enhanced the ability to address all movement ecology questions [77], the technology has not advanced enough for improving information on smaller-bodied species incapable of carrying those packages. Studying dispersal in wild populations of small bodied animals is challenging due to the spatiotemporal scale, particularly for migratory species that move long distances to and from nonbreeding areas between breeding seasons, that is needed to track marked individuals coupled with the geographical limits of study sites, temporal limitations of studies, and difficulties in disentangling the effects of individual and ecological factors for a particular system [11, 35, 45, 61, 67]. Consequently, empirical studies on dispersal distances in wild populations has received even less attention compared to research on dispersal probabilities [67]. However, what follows dispersal (i.e., how far individuals move, where to settle, how successful subsequent breeding attempts are) may have different drivers than dispersal probabilities, important fitness consequences for individuals, and lasting effects on population structure [23, 67]. Using a movement ecology framework to evaluate dispersal distance therefore means to evaluate why, how, when, and where individuals move different distances [46].

Dispersal is a nonrandom movement that is influenced by an individual’s social and physical environment and many factors alter the cost–benefit balance of dispersal patterns [18, 39, 67]. Natal dispersal has evolved to reduce competition and inbreeding and is the primary mechanism for maintenance of genetic population structure [48]. Adults that disperse between breeding attempts may ultimately enhance their fitness when moving to a new breeding territory in order to increase access to mates or to higher-quality habitats that have fewer predators or competitors [13, 23]. Individuals may disperse following either an unsuccessful reproductive attempt or loss of a mate in order to improve future reproductive success [44, 66, 76]. Furthermore, environmental conditions may interact with individual characteristics to influence either natal or adult dispersal decisions [18, 31]. Dispersal costs generally increase with longer dispersal distances, due to energetic consequences and increased predation risk related to movement to a novel habitat [11, 43, 59, 67]. Therefore, individuals are only expected to move long distances if the benefits outweigh costs, such as decreased predation, resource limitation, or conspecific competition [21, 33, 42]. Changes to previously used nesting areas such as a disturbance event (e.g., flooding) or an increase in competition may increase dispersal distances by decreasing the realized quality of a territory in terms of resource abundance, availability, and/or distribution [33, 34]. Moreover, dispersal movements may be short, such as moving to an adjacent territory, or relatively far, which could signify emigration to another subpopulation. Dispersing to alternate breeding areas therefore involves a series of movement decisions regarding whether to disperse (i.e., why), when and how (i.e., what path) to do so, and how far and where to go—all of which will be influenced by multicausal processes.

Dispersal affects the potential for colonization of new favorable habitats, range expansion, and gene flow, making it a critical factor in conservation planning. Breeding dispersal distances tend to be shorter for abundant generalist species, and longer for species that specialize on patchily distributed habitats [38, 48]. The abundance, availability, and distribution of resources and threats across the landscape matrix likely also influence the amount of time and distance required to locate a new suitable site and successfully breed [33, 50]. Successful conservation strategies must consider movements of individuals of multiple age classes and their ability to disperse to available nesting sites, particularly in fragmented landscapes or where habitat is patchy [1, 19]. Therefore, understanding dispersal distance is critical for identifying the locations of potential conservation sites for species conservation and planning.

An increased understanding of which environmental and social factors affect dispersal distance in declining populations can help inform conservation. The piping plover (*Charadrius melodus;* hereafter ‘plover’) is a small migratory shorebird endemic to North America with breeding populations in the Atlantic Coast, Great Plains, and Great Lakes regions. The species was Federally listed due to concerns over habitat loss and low reproductive output [73, 74]. Plovers in the Northern Great Plains rely on breeding habitat with little to no vegetative cover on riverine sandbars, reservoir shorelines, or dry margins of wetlands in the Prairie Pothole Region commonly referred to as alkali wetlands [3, 5, 53, 71]. Individual plovers have been documented making long distance dispersals of ~ 1500 km between major breeding populations [26, 29]. However, mean natal dispersal distances were much shorter (males: 8.6 ± 16 km, females: 12.8 ± 24.5 km) as were breeding dispersal distances (males: 35 ± 14.5 km; females: 26 ± 9.8 km) [2, 15, 26], which may limit the ability for individuals to discover available nesting sites. Dispersal probabilities are higher following years of poor reproductive success and following flood years [55, 56], suggesting that prior reproductive success, mate fidelity [24], and environmental factors may influence not only the decision to disperse but also the distance individuals move. Piping plovers are monitored throughout their breeding range, providing a unique opportunity to use observations of marked individuals to evaluate dispersal distances as their movements outside of a core study area still have a high probability of detection.

Our primary objective was to examine the causes of variability in piping plover natal dispersal and adult interannual breeding distances, particularly in relation to habitat availability, local conspecific density, and reproductive success (Table 1). We predicted that dispersal distance would increase with decreasing habitat availability (Table 1). We also examined the effects of local density (current and prior), current reproductive success (nest initiation date, mate fidelity, and hatching success), and proximity to alternative breeding areas on interannual breeding distance. We predicted that movements would be longer for individuals in high density areas, for later initiated nests, and when alternative breeding sites were farther away (Table 1). Using banding data for a species monitored throughout its range, we aimed to understand the causes and spatiotemporal patterns of individuals that relocate to breed [46].

**Table 1:** A priori hypotheses for variables affecting natal dispersal and interannual adult breeding movement distances

Explanatory variable	Age class	Type	A priori hypothesis
Estimated hatch date	Natal	Individual	Individuals hatched later in the breeding season will disperse shorter distances
Nest initiation date at settled site	Adult	Reproductive success	Individuals will start nests later after longer breeding dispersal movements
Previous or natal habitat type	Both	Habitat	Individuals hatched on or previously bred on reservoirs will disperse longer distances
Settled habitat type	Both	Habitat	Individuals settling on river habitat will move the shortest distances
Available habitat index at previous or natal site	Both	Habitat	When more habitat is available at the previous or natal site, individuals will move shorter distances
Available habitat index at settled site	Both	Habitat	When more habitat is available at the settling site, individuals will move farther distances
Chick density	Natal	Social	Sites with high densities of chicks will have increased natal dispersal distances
Adult density at previous site	Adult	Social	Sites with high densities of adults will have increased movement distances
Adult density at settled site	Both	Social	Individuals will move farther distances to nest with more conspecifics
Mate fidelity at settled nest	Adult	Individual	Retaining a mate between consecutive nesting attempts will decrease the distance between nesting attempts
Reproductive success at previous site	Adult	Reproductive success	Individuals with unsuccessful nesting attempts will move farther between nests
Reproductive success at settled nest	Adult	Reproductive success	Individuals that moved farther between nest attempts will have better reproductive success
Average proximity to other nesting areas of settled nest	Both	Habitat	Farther distances between nesting areas will increase breeding movement distances

## Materials and methods

### Study area

**Fig. 1:**
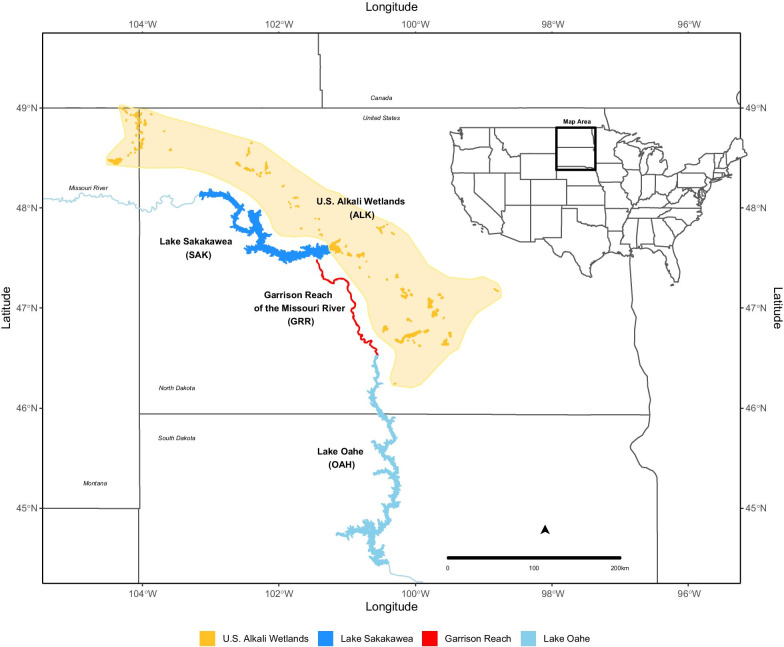
Piping plover nesting areas in North Dakota, South Dakota, and Montana from 2014 to 2019. The four management units studied are shown: Lake Sakakawea, U.S. Alkali Wetlands, Garrison Reach of the Missouri River, and Lake Oahe

From 2014 to 2019, we studied breeding piping plovers on alkaline wetland, reservoir, and riverine nesting habitats from central South Dakota through North Dakota and into northeastern Montana, USA (Fig. 1). Our project included four management units: the U.S. Alkali Wetlands, Lake Sakakawea, the Garrison Reach of the Missouri River, and Lake Oahe (see [71] for details on management units). The Alkali Wetlands region consisted of ~ 150 basin (i.e., lake, pond, or slough) shorelines located on public and private lands in the Missouri Coteau ecoregion of North Dakota and Montana. Reservoir habitat consisted primarily of mainland and island shorelines along Lake Oahe and Lake Sakakawea (two mainstem reservoirs of the Missouri River) as well as several reservoir-like wetland basins with water management systems (e.g., Lake Audubon—Audubon National Wildlife Refuge (NWR), Medicine Lake—Medicine Lake NWR, Long Lake—Long Lake NWR, Jim Lake—Arrowwood NWR). The riverine habitat consisted of sandbars on the Missouri River’s Garrison Reach, which extended from the Garrison Dam to the headwaters of Lake Oahe in North Dakota.

Compared to most previous studies of individually marked birds, our study area covered a much larger spatial scale encompassing the majority of available habitat across three states. Concurrently, plovers breeding on the southern Missouri, Platte, and Niobrara Rivers, as well as the other two breeding populations, were monitored by other agencies and citizen birders who reported band resights and nest locations to us if our marked birds were observed. We acknowledge plovers could have nested in alkaline wetlands in Canada and in the United States that were not monitored; therefore, we may have failed to detect some long-distance movements. All field studies will have some spatial limitation, but we argue that the size of our study area, particularly with concurrent work in other breeding areas, mitigates most of this risk.

### Field methods

Each year, from late April to early August, crews searched appropriate habitat or used behavioral observations to locate plover nests, chicks, and adults. The area searched varied each year due to habitat availability, but field crews monitored any area where plovers were seen [71]. Using a combination of grid-searches and searches based on plover behavioral cues, crews searched sandbars and shorelines for nests. Once located, nests were monitored until completion (i.e., until all eggs either hatched or nests were determined to have failed, see [64] and [6] for more detailed discussion). For each nest, we collected data on the location, nest habitat, estimated date of hatch or failure, and identities of incubating adults.

We have banded plovers with a U.S. Geological Survey metal band and unique alpha-numeric engraved flag since 2013 on the Garrison Reach and Lake Sakakawea and since 2014 on Lake Oahe and the Alkali Wetlands. Banding efforts concluded at the end of the 2017 field season, but we continued resighting banded plovers through 2019. We trapped unbanded adult plovers on nests during incubation using either a modified remote-controlled walk-in trap or bow-net [57]. Individuals were attributed to nests by capturing individuals on the nest, observing an identified individual return to incubate, or using high-definition video cameras set up near (45–60 cm away) nests for no more than 30 min at a time (Kodak PixPro spz1 video cameras [72]). Chicks were banded in the nest bowl when possible, if chicks were older and more mobile, they were captured by hand or with butterfly nets, banded, and attributed to nests by the identities of attending adults.

### Individual covariates

We estimated nest initiation and hatch dates using two methods, depending on the availability of certain types of data. If chicks were observed in the nest bowl, the primary method for estimating initiation date was backdating it from the observed hatch date (assuming laying and incubation period of 35 days). If hatch date was unknown, we estimated hatch date by adding 35 days to the estimated initiation date. We estimated initiation date (NID) from incubation stage using egg floatation [37] using the visit date the nest was discovered (visit), the number of eggs at nest discovery (eggs), the incubation stage at nest discovery (stage) and the following formula:

$${\text{NID}} = {\text{visit}} - \left( {\left( {{\text{eggs}} - {1}} \right)*{2}} \right) - {\text{stage}}$$

For adults, we categorized mate fidelity as a three-level factor: retained mate from previous year (both mates known in both years), divorced mate from previous year (both mates known in both years), and unknown if in one or both years their mate was unknown or was unbanded. We also defined nest fate into the following categories: successful (at least one egg hatched), failed during laying or incubation, or unknown/not fully monitored.

Habitat type was treated as a three-level factor: reservoir, river, or alkali wetland. Management units were subdivided into segments of potential nesting habitat, which were defined differently for each habitat type. For river habitat, a segment was a predefined section of equal length (one river mile) which included stretches of sandbar and shoreline habitat as well as flowing water. On the reservoirs, previously described segments of roughly 2 km reservoir shoreline based on the 2004 pool elevation (see [4]) were re-measured as the perimeter length at the maximum pool elevation of the reservoir shoreline. On alkali wetlands, a segment was the perimeter of the wetland itself, derived from the National Wetland Inventory polygons.

Adult density was calculated as twice the number of nests found on a segment corrected for known renesting probabilities [68] during that breeding season divided by the length of that segment because conspecific abundance can influence nest survival [69]. Because nest monitoring efforts varied among years of study, we estimated chick density as the number of chicks hatched from nests on each segment in each year with a series of assumptions. First, if chicks were found in the nest bowl, we used the number of chicks found. If a nest was presumed successful due to alternate pieces of evidence (e.g., chick tracks, droppings, or pipping fragments [68]), we used the clutch size corrected for hatching rate of eggs (78.5%, [71]) estimated from 129 successful nests closely monitored from 2014 to 2015. We standardized chick and adult densities within each management unit due to the differences in segment length measurement strategies.

To assess how habitat availability may drive dispersal distances, we calculated a standardized index of habitat availability and derived the change in available nesting habitat from the year prior to the year of interest to represent the dynamic availability of habitat more accurately. We calculated the index of habitat availability differently for each habitat type. For wetland basins with water management systems (those that act as reservoirs), we used the maximum water elevation measures for each month at gauges monitored by the U.S. Fish and Wildlife Service. For all other alkaline wetlands, we represented habitat with an index of climate that was developed specifically for hydrological effects of climate on permanent and semi-permanent wetlands of the Prairie Pothole Region [52]. This index is based on the Standard Precipitation–Evapotranspiration Index (SPEI, [10]) but is calculated using a 72-month average from monthly PRISM (Parameter-elevation Regressions on Independent Slopes Model) data from the PRISM Climate Group (Oregon State University, Corvallis, Oregon, USA) because that time frame has been demonstrated useful for predicting water level dynamics in wetlands of similar size and hydroperiod [40, 52]. We calculated a site-specific index of habitat change by subtracting SPEI values for May, June, and July and selecting the maximum change from the year prior to the year of interest within the breeding season. Negative values indicate drier conditions and subsequent drawdown of water level and an increase in availability of nesting habitat [41, 58]. For Garrison Reach, we used the change in maximum monthly Garrison dam outflow (1,000 cubic feet per second) between May and July from the prior year to the focal year (data available from U.S. Army Corps of Engineers: http://www.nwd-mr.usace.army.mil/rcc/information). Positive values indicate that dam outflow increased during the breeding season, reducing available nesting habitat. For reservoirs, we used a predictive model on the amount of available plover habitat developed for Lake Sakakawea [7] and adapted for use on Lake Oahe, which takes into account elevation, vegetation growth, and ice scour.

Lastly, we developed a measure of the proximity to other breeding areas to account for the patchy availability of habitat. For each nest location, we calculated the mean Euclidean distance to the three nearest known nests on different segments during that year using the spatstat package v.1.64 [8].

All data generated during this study are publicly available as a USGS data release [70].

### Statistical analyses

We calculated Euclidean dispersal distance as either a) the distance between the nest a chick was hatched from to the first known nest that individual bred in (natal dispersal) or b) the distance between two successive breeding attempts for adults (interannual adult breeding movement) (package sp; [49]). Because plovers do not defend stable territories between years, we assumed that distances shorter than 50 m between successive breeding attempts represented philopatric movements because the adults were likely using the same space in both years, and we removed them from our analysis of adult breeding movements. We, therefore, included all successive breeding attempts > 50 m in our analyses even when movements were otherwise short for adult interannual breeding movements. Thus, we define interannual breeding movements to include all movements > 50 m by individuals between known nesting attempts. Assignment of individuals to a nest is not perfect, and plovers can forgo breeding in some years [17, 71]. So, we included instances where there was a gap year between breeding locations (i.e., when individuals were not attributed to a nest location).

We investigated variation in dispersal distance in response to our a priori hypotheses on environmental, individual, social, and reproductive success factors (Table 1). We examined sources of variation in dispersal distances using two (natal and adult) global generalized linear models (GLM) with a Gamma distribution in R statistical software (3.5.0; R Development Core Team 2018; package lme4; [9]). We first checked for correlations among the independent variables and reduced variables as needed (if |r| > 0.6, all remaining correlations were below |r| = 0.3). All covariates included in the final global models are listed in Table 1. We assessed model fit by examining plots of the observed versus predicted points and residuals. To ensure model convergence and interpretability of beta estimates, all covariates were standardized to a mean of 0 and a standard deviation of 1, except for the categorical covariates. The α dispersion coefficient was estimated with the MASS package [75] in program R. We then calculated profile confidence intervals and evaluated the significance of each parameter of interest in the fitted global GLM for each response variable. We considered effects to be strongly supported if the 95% confidence interval (CI) for the parameter coefficient did not include zero.

Our dataset for adult interannual movements contained multiple movements from some individuals; however, models failed to converge when we included a random effect of individual. When we used only the data for individuals with more than one movement, the interclass correlation coefficient for distance was 0.38 (95% CI 0.32–0.43), indicating low measurement repeatability [78], so we retained all movements by individuals in our analyses.

## Results

### Natal dispersal distance

**Fig. 2:**
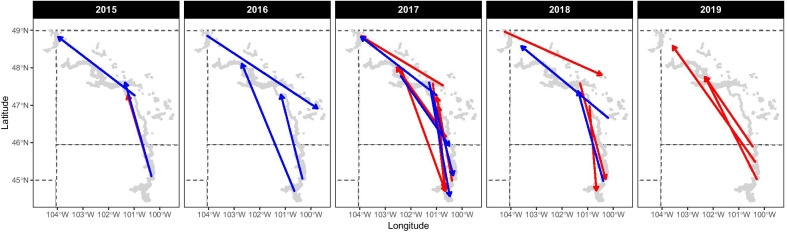
Natal and adult piping plover movements that exceeded 250 km but remained within our study area. Natal movements are shown in blue, and adult movements are shown in red. Arrows indicate direction from previous or hatching nest location to breeding location in year of grid. For example, an adult moved from Lake Oahe (South Dakota) to breed on Garrison Reach (North Dakota) in 2015 (left, red arrow)

Dispersal distances of piping plovers between their natal nest and the location of their first known breeding attempt varied widely (mean: 81.0 km, median: 53 km, range 1–410 km, CV: 93.8). We observed 275 natal dispersal events within our study area (Table 2). Despite banding over 2669 individuals over four cohorts (38% on reservoir, 39% on river, and 24% on alkali wetland habitats), roughly half (48%) of our observed natal dispersal events originated on the river habitat (32% from reservoirs and 20% from alkali wetlands). Eighty-eight percent (88%) of individuals dispersed over 10 km, and 33% of individuals dispersed over 100 km. Fifty-three percent (53%) of individuals were known to breed for the first time in their first year after hatch. Three additional individuals left our study area to breed in the southern Missouri River segments or on the central Platte River in Nebraska and were not included in our analysis. Within our study area, two individuals dispersed more than 350 km: one from Lake Oahe to Lake Sakakawea and one from a northwestern alkaline wetland to the Garrison Reach. An additional nine individuals moved more than 250 km (Fig. 2). Of the observed movements within our study area, 42% of individuals dispersed away from their natal management unit.

**Table 2:** Number of natal dispersal and interannual breeding movements and mean (± standard deviation) distances

Start location	End location	Natal dispersal			Adult breeding movements		
		#	Median distance (km)	Mean (± SD) distance (km)	#	Median distance (km)	Mean (± SD) distance (km)
U.S. Alkali Wetlands	U.S. Alkali Wetlands	41	48.4	80.3 (92.2)	160	2.3	15.7 (37.5)
	Lake Sakakawea	15	129.7	118.1 (50.4)	4	114.0	119.3 (52.6)
	Garrison Reach	6	94.1	92.8 (40.3)	6	50.8	58.0 (28.7)
	Lake Oahe	7	164.1	159.2 (34.4)	3	213.6	205.4 (111.8)
Lake Sakakawea	U.S. Alkali Wetlands	16	77.2	88.7 (71.2)	43	52.7	77.9 (58.1)
	Lake Sakakawea	16	42.0	43.3 (31.8)	201	1.8	11.8 (22.0)
	Garrison Reach	5	103.2	92.8 (36.7)	35	92.0	87.7 (43.5)
	Lake Oahe	7	238.1	239.0 (61.7)	7	242.4	255.2 (64.2)
Garrison Reach	U.S. Alkali Wetlands	12	106.9	113.2 (78.8)	52	88.0	82.6 (31.4)
	Lake Sakakawea	36	97.9	99.8 (46.5)	29	94.9	96.1 (50.3)
	Garrison Reach	80	23.5	28.1 (19.3)	592	1.2	11.1 (19.6)
	Lake Oahe	4	170.8	153.2 (41.8)	27	94.0	128.0 (71.5)
Lake Oahe	U.S. Alkali Wetlands	4	157.3	149.9 (29.5)	6	170.1	198.2 (120.8)
	Lake Sakakawea	4	265.6	295.0 (79.8)	7	233.6	235.2 (55.6)
	Garrison Reach	6	138.5	164.1 (87.3)	20	145.7	146.2 (63.1)
	Lake Oahe	16	27.1	38.8 (39.3)	143	1.4	11.9 (26.2)

Natal and adult interannual breeding movements (> 50 m) for piping plovers in the Northern Great Plains during 2014–2019

**Fig. 3:**
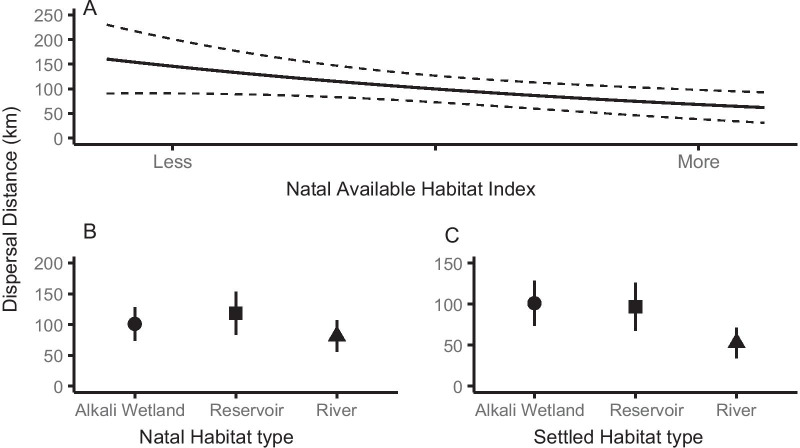
Habitat influences on natal dispersal distance. Effects of **a** change in available habitat index at the natal area, **b** natal habitat type, and **c** adult breeding area habitat type on natal dispersal distance (km) of Northern Great Plains piping plovers during 2014–2019. Dotted lines and error bars indicate 95% confidence intervals

Natal habitat type, natal available habitat, and breeding location habitat type all influenced natal dispersal distance. Natal dispersal distances decreased when more habitat was available on their natal area than in the year prior (β =  − 0.18; CI [− 0.33, − 0.04]; Table 3, Fig. 3a). Individuals hatched on the river habitat dispersed the shortest distances while those hatched on reservoirs dispersed the farthest (Table 3, Fig. 3b). Individuals that settled to breed on river habitats for their first breeding attempt dispersed shorter distances than those that settled on alkali wetlands or reservoirs (Table 3, Fig. 3c).

**Table 3:** Parameter estimates, standard errors (SE), and 95% confidence intervals (CI) from fitted models

Parameter	Natal dispersal distance			Adult interannual breeding distance		
	β	SE	95% CI	β	SE	95% CI
Intercept	**4.51**	**0.14**	**(4.23, 4.80)**	**45.33**	**6.22**	**(33.14, 57.52)**
Estimated hatch date	0.03	0.06	(− 0.10, 0.16)	–	–	–
Nest initiation date at settled site	–	–	–	**2.79**	**0.64**	**(1.54, 4.04)**
Previous habitat type – RESERVOIR	**4.68**	**0.15**	**(4.41, 4.97)**	**21.07**	**4.27**	**(12.70, 29.43)**
Previous habitat type – RIVER	**4.26**	**0.16**	**(3.99, 4.55)**	-2.50	2.10	(− 6.62, 1.62)
Settled habitat type – RESERVOIR	− 0.08	0.16	(− 0.22, 0.37)	**− 13.69**	**4.49**	**(− 22.49, − 4.88)**
Settled habitat type – RIVER	**− 0.56**	**0.17**	**(− 0.85, − 0.27)**	**− 16.93**	**4.80**	**(− 25.70, − 8.15)**
Previous index of available habitat	**− 0.18**	**0.07**	**(**− **0.33, **− **0.04)**	1.88	1.08	(− 0.24, 4.01)
Settled index of available habitat	0.05	0.07	(− 0.09, 0.19)	**3.62**	**0.59**	**(2.47, 4.77)**
Natal chick density	− 0.06	0.06	(− 0.18, 0.09)	–	–	–
Previous adult density	–	–	–	0.16	0.42	(− 0.69, 1.00)
Adult density at settled site	− 0.03	0.05	(− 0.12, 0.07)	0.10	0.38	(− 0.64, 0.85)
Mate fidelity at settled nest – RETAINED	–	–	–	**− 7.77**	**1.35**	**(− 10.40, -5.13)**
Mate fidelity at settled nest – UNK	–	–	–	− 2.07	2.05	(− 6.09, 1.96)
Nest fate at settled nest – HATCHED	–	–	–	− 2.12	2.57	(− 7.16, 2.92)
Nest fate at settled nest – UNK	–	–	–	3.36	2.66	(− 1.85, 8.57)
Previous nest fate – HATCHED	–	–	–	**− 15.35**	**4.09**	**(− 23.36, − 7.33)**
Previous nest fate – UNK	–	–	–	**− 11.98**	**4.13**	**(− 20.09, − 3.88)**
Average proximity to other nesting areas at settled nest	0.09	0.05	(− 0.01, 0.19)	**2.82**	**1.42**	**(0.03, 5.60)**

Models are for natal and adult breeding movement distances of piping plovers in the Northern Great Plains during 2014–2019. Significant variables (where 95% CI did not cross zero) are bolded. Previous is natal site for natal dispersal distance

### Adult interannual breeding distance

We observed a total of 1,709 movements from 994 individuals. Adult movement distances varied widely (mean: 23.7 km, median: 0.95 km, range: 0–816 km, CV: 227.0) and may include some philopatric individuals as we do not have data on plover territories. After eliminating movements < 50 m, 1,335 adult movements within our study area remained for 878 individuals (mean: 28.5 km, median: 3.7 km, range 0.05–422 km; Table 2). In total, 73% of our interannual breeding movements had no gap year between nest locations. An additional four individuals dispersed over 500 km either to or from our main study area and breeding areas on the central Platte River in Nebraska, which were not included in our analysis. The longest dispersal event within our study area was from Lake Oahe to a northwestern alkaline wetland (422 km; Fig. 2). Fifteen individuals moved over 250 km within our study area (Fig. 2). One individual dispersed between different management units four times during the study, and 13% of individuals dispersed to a different management unit. Twice, individuals retained a previous mate while dispersing between management units. In 2018, a pair dispersed to the Garrison Reach after successfully hatching a nest together on Lake Sakakawea in 2017. Two individuals that had bred together on Lake Oahe in 2015 divorced and subsequently both dispersed (seen nesting separately during the interim) and reunited to pair and breed in a central alkali wetland in 2019.

**Fig. 4:**
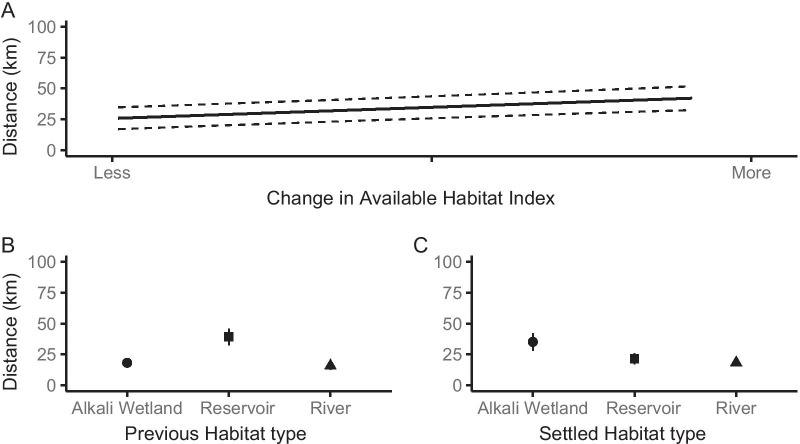
Habitat influences on adult interannual breeding distance. **a** Effects of the change of habitat available at settled site, **b** habitat type of the dispersed (previous) site, and **c** habitat type at the settled site on adult interannual breeding distance (km) of Northern Great Plains piping plovers during 2014–2019. Dotted lines and error bars indicate 95% confidence intervals

**Fig. 5:**
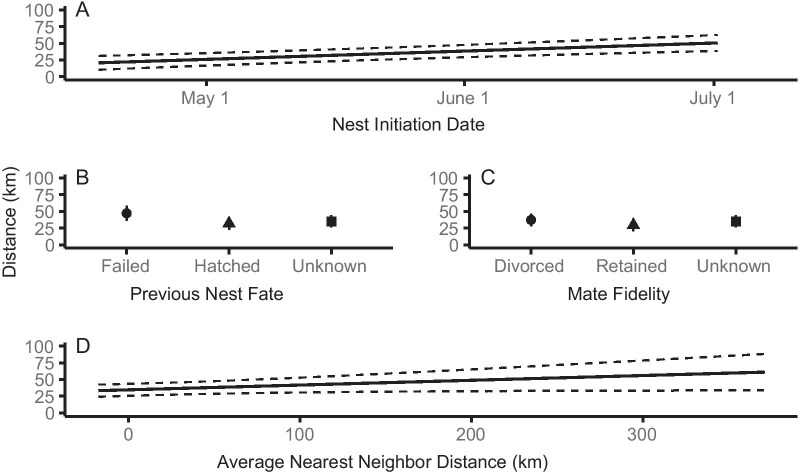
Reproductive success and habitat proximity influences on adult interannual breeding distance. Adult breeding movement distance (km) of Northern Great Plains piping plovers varied with **a** nest initiation date of the settled nest, **b** previous year’s nest success, **c** mate fidelity at the settled nest, and **d** proximity to other nesting areas of the settled site. Dotted lines and error bars indicate 95% confidence intervals

Adult interannual breeding distance was associated with habitat type and availability as well as individual-level reproductive success. Individuals moved farther distances if there was more habitat available in the settling year than the previous year (β = 3.62; CI [2.47, 4.77]; Table 3, Fig. 4a). Breeding previously on river habitat also shortened distances compared to reservoir habitats (Table 3, Fig. 4b). Individuals settling on river habitats to breed dispersed the shortest distances while those settling on alkali wetlands dispersed the farthest (Table 3, Fig. 4c). Longer distances were associated with later nest initiation dates on the settled site (β = 2.79; CI [1.54–4.04]; Table 3, Fig. 5a). Individuals whose nests failed in the previous year’s reproductive attempt moved longer distances, a nest successfully hatching in the previous year shortened movement distances (Table 3, Fig. 5b). Individuals that retained their mate from the previous year moved shorter distances (Table 3, Fig. 5c). Lastly, distances were shorter when settled sites were in closer proximity to other breeding areas (β = 2.82; CI [0.03, 5.60]; Table 3, Fig. 5d).

## Discussion

We found that habitat type and habitat availability were associated with both natal dispersal and adult interannual breeding distances for piping plovers. Natal dispersal was influenced by habitat type at both natal and settled sites and habitat availability at the natal site. Adult interannual breeding distances varied with habitat type at the previous breeding and settled breeding sites and habitat availability at the settled site. As predicted, individuals dispersed shorter distances when more habitat was available (natal site for natal dispersal and settled breeding site for adult interannual movements). When alternative breeding areas were closer to settled nesting sites, adults moved shorter distances between breeding attempts. Individuals of both age classes also dispersed the shortest distances when leaving or settling on the river habitat. Further, adult interannual breeding distances increased with failed previous reproductive attempts, later nest initiation dates at the settled site, and a lack of mate fidelity. However, we found no effect of local conspecific densities at either site on natal or adult dispersal distances. Overall, our results show that both environmental conditions and individual reproductive success influence interannual breeding distance, both of which will have strong fitness consequences, and natal dispersal distance was primarily associated with habitat type and availability. Since habitat availability is predicted to decline in the alkali wetlands [41], plovers may have to increase dispersal distances to find suitable nesting habitat. Increased dispersal distances should be of concern for conservation efforts because changing dispersal behavior may affect population vital rates such as higher rates of mortality and/or emigration to other subpopulations.

Animals occupying habitats with unstable conditions typically show higher dispersal or longer movements between alternate breeding sites [25]. Plovers utilize early successional habitat for nesting, and on the Missouri River are dependent on floods or wave- and ice-scour to remove or prohibit the growth of vegetation and create suitable nesting habitat. Historically, seasonal water level fluctuations maintained early successional habitat conditions on prairie rivers, where peak flows in March and June submerged existing sandbars and redistributed sediments, creating unvegetated sandbars suitable for plover nesting as water levels receded [14, 16]. In the absence of high natural flows, conservation activities have maintained vegetation-free sandbars through vegetation removal or the construction of sandbars [16, 65]. While the Missouri River is hydrologically linked and can show high spatiotemporal autocorrelation in habitat availability in some years, particularly when water levels are high and little habitat is available, the Prairie Pothole Region experiences frequent fluctuations in precipitation that vary within the region, increasing water level variability among wetlands with different wetlands experiencing different water levels at the same time [41, 52]. Therefore, along the Missouri River, and in particular the northern river segments, habitat is fairly continuous spatially, though may be temporally variable based on releases from the upstream dams. Unlike reservoir or alkali wetland habitats, the sandbars on the Garrison Reach provide a centrally located, semi-continuous corridor of nesting habitat. Individuals hatched on, previously nested on, or settling on the Garrison Reach moved the shortest distances. Individuals on the Garrison Reach had higher daily nest survival, daily chick survival, renesting probabilities, renest reproductive success, and apparent annual survival compared to individuals on the reservoirs [6, 68]. Our analyses also suggest that river habitats may be considered high quality as individuals generally moved short distances when dispersal occurred.

We found that habitat availability influenced natal dispersal and adult interannual breeding distances in different ways. Natal dispersal was influenced by changes in habitat availability at the natal area, whereas adult movements were best explained by habitat availability at the settling site. As the availability of habitat (at the natal location for hatch-year birds and at the settled location for adults) increased, individuals moved shorter distances, confirming our predictions. Further, adults dispersed shorter distances when the average distance was shorter to the closest three nesting areas. This suggests that individuals seek out alternative nesting habitats near previous nesting sites and that the spatial configuration of available habitat affects movements of individuals. Similarly, natal dispersal probabilities in Dunlin (*Calidris alpina*) decreased with increasing natal patch size and increasing distance to alternative patches [47]. Habitat-driven dispersal is not surprising for plovers, which rely on early-successional habitats for nesting [4], but most individuals in both age classes dispersed short distances between breeding attempts suggesting individuals rely on a complex of nearby sites when searching for alternative nesting areas. The spatial structure of available habitat may therefore have important consequences for population dynamics and conservation planning as habitat may limit movements and gene flow between disparate breeding areas.

While the mean distance moved was longer for hatch-year birds, adults showed more variability and low repeatability in movement distances. Contrary to the long-standing view that the longest dispersal movements occur prior to an individual’s first breeding attempt [25], adults made the longest documented movements in this system (nearly double that of natal dispersal). Ortolan Buntings *(Emberiza hortulana),* which breed in patchy habitat, also exhibit short natal dispersal movements and longer adult breeding dispersal [20]. Adults may be more discerning than first-time breeders in choosing a settling site to ensure current reproductive success either by improving the quality of their territory or mate compared to their previous attempts leading to more variation in movement lengths due to the patchy availability of habitat. It may be advantageous for individuals to return near their natal area to breed to improve their chances of reproduction in their second year, and if unsuccessful, search for more distant sites later in life using conspecifics as a cue for high quality habitat patches [20, 54, 55]. Thus, there may be an advantage for individuals to maintain flexibility in dispersal distances when the availability of habitat is patchy.

Conspecific densities during the breeding season can confer varied impacts on plover reproduction, including rates of double-brooding [30] and nest survival [69]. While density dependent dispersal is thought to be due to limitations in available habitat, we did not detect a relationship between dispersal distances and densities at the hatching, previous, or settling sites for either age class. Adult plovers have been shown to use public information to select nesting sites when there is interannual variation in habitat quality [55]. The lack of support for a relationship between natal dispersal distance and chick or adult densities may indicate that first-time breeders are constrained to lower-quality nesting habitats because of intraspecific competition, or alternatively that the use of conspecific cues when selecting nesting sites is a learned behavior. However, we also did not detect an effect for adults. Our results differ from previous findings from other study areas [15, 55], therefore plover dispersal distances may be mediated by density dependent processes in some, but not all habitats. Density estimates based on the amount of habitat (like those used here for the reservoirs but were unavailable for alkali wetland or river habitat types) might further enlighten this relationship. Indeed, an international piping plover census indicated that plovers do not use all apparently suitable habitats within their geographic range [51], suggesting that a better understanding of the factors contributing to habitat quality is still needed.

Previous and current reproductive success influenced interannual breeding distances for adult plovers. As we predicted, dispersal distance increased for plovers that experienced hatching failure the previous year and those that divorced their mate. Previous reproductive success can influence dispersal probabilities in plovers [55, 56], as would be expected as dispersal is only beneficial if fitness increases. Individuals that have low reproductive success presumably attempt to disperse to an area of higher quality the following year to increase reproductive output [36, 62], and individuals may divorce their partners to improve reproductive success [27]. Nests on alkali wetlands during this same period of time had higher survival rates compared to river or reservoir habitats [68]. Plovers dispersed the farthest when settling to breed on alkali wetlands. While this could be due to the inherent dispersed nature of alkali wetland habitat, individuals could also move to alkali wetlands to improve future reproductive success. However, longer dispersal distances may still retain some costs for plovers. Individuals that moved farther initiated their current nest later in the breeding season, likely a manifestation of some immediate travel cost to individuals or lack of familiarity with a novel territory. Delayed breeding suggests that long-distance dispersal may have fitness consequences as daily nest survival declines later in the breeding season for plovers in all three habitat types [68]. This study did not address other potential costs, such as reduced survival, which could also reduce potential fitness benefits of dispersing farther.

Our study adds to the growing knowledge about dispersal distances in piping plovers. Collectively, breeding movements were skewed toward short-distance dispersal events with the median distance (1 km) indicating that most adults do not move far between successive breeding attempts. However, we detected 15 adult breeding movements and 11 natal dispersal events over 250 km that remained within our focal study area (Fig. 2) and 133 adult and 33 natal dispersal events over 100 km. Similar dispersal events over 250 km have been documented in the Canadian Atlantic [2] and Lake of the Woods, Minnesota [26] breeding populations, with maximum single individual events of 1200 km (adult [26], and 1500 km (juvenile,[29]. Isolated subpopulations, such as Lake of the Woods, and discontinuous habitat over a broad area, like the Gulf of St. Lawrence, seem to lead to longer dispersal events. In contrast, our study area covers roughly 84,000 km^2^ of both semi-continuous (Missouri River) and discrete (wetland) habitat, yet individuals routinely dispersed amongst habitat types and over long distances suggesting that connectivity within this region is high. While this study examines what influences movement lengths, further work focusing on dispersal probabilities may better aid our understanding of the implications of long-distance movements on individual survival and population structure.

## Conclusions

Piping plovers are capable of long-distance dispersal between breeding populations, yet these long-distance events are relatively infrequent. In this study, we did not document any movements between breeding populations but did show small numbers of individuals moving from the Northern Great Plains to the southern segments of the Missouri River and to the Platte River. However, we did find frequent long-distance dispersal within the northern segments of the Missouri River and the U.S. Alkali Wetlands suggesting not only high connectivity within this region but also a dynamic landscape where individuals respond to the availability of habitat across both space and time. Our findings suggest a potential for compounding implications for local areas being managed to maintain a targeted abundance of adult plovers, such as is done on the Missouri River [73], because flooding or predation reduces recruitment, but lower productivity also increases the distance adults move, potentially off the targeted system, making local estimates unreliable. This suggests that management for habitat quality, that is habitat that can produce fledglings, is perhaps more important than previously thought for maintaining a target of returning adults. Habitat type and availability were important variables explaining variation in dispersal distances for both hatch-year and adult plovers. Therefore, conservation efforts should encompass multiple scales to account for dispersal distances that range widely (< 1 km to > 400 km) and dynamic habitat availability between both the Missouri River and the Alkali Wetlands. Maintaining a network of sites including complexes of nearby (1 km) and distant (> 50 km) breeding sites would be beneficial for plovers to accommodate changes in habitat availability especially as habitat becomes more limited in the future.

## Data Availability

Data available as a USGS data release https://doi.org/10.5066/P96PSOBQ [70].

## Abbreviations

Plover: Piping plover
NWR: National wildlife refuge
SPEI: Standard precipitation-evapotranspiration index
PRISM: Parameter-elevation regressions on independent slopes model
GLM: Generalized linear model
CI: Confidence interval
